# Thermostable Cellulase Biosynthesis from *Paenibacillus alvei* and its Utilization in Lactic Acid Production by Simultaneous Saccharification and Fermentation

**DOI:** 10.1515/biol-2020-0019

**Published:** 2020-04-10

**Authors:** Yasser S. Mostafa, Saad A. Alamri, Mohamed Hashem, Nivien A. Nafady, Kamal A.M. Abo-Elyousr, Zakaria A. Mohamed

**Affiliations:** 1 King Khalid University, Faculty of Science, Biology Department, Abha Saudi Arabia; 2 Prince Sultan Bin Abdulaziz Center for Environmental and Tourism Research and Studies, King Khalid University, Abha Saudi Arabia; 3 Assiut University, Faculty of Science, Botany and Microbiology Department, Assiut, Egypt; 4 Assiut University, Faculty of Agriculture, Plant pathology Department, Assiut, Egypt; 5 King Abdulaziz University, Faculty of Meteorology, Environmental and Arid Land Agriculture, Department of Arid Land Agriculture, Jeddah Saudi Arabia

**Keywords:** *Paenibacillus alvei*, Cellulase, Date palm leaves, Lactic acid, SSF

## Abstract

Cellulosic date palm wastes may have beneficial biotechnological applications for eco-friendly utilization. This study reports the isolation of thermophilic cellulase-producing bacteria and their application in lactic acid production using date palm leaves. The promising isolate was identified as *Paenibacillus alvei* by 16S rRNA gene sequencing. Maximum cellulase production was acquired using alkaline treated date palm leaves (ATDPL) at 48 h and yielded 4.50 U.mL^-1^ FPase, 8.11 U.mL^-1^ CMCase, and 2.74 U.mL^-1^ β-glucosidase. The cellulase activity was optimal at pH 5.0 and 50°C with good stability at a wide temperature (40-70°C) and pH (4.0-7.0) range, demonstrating its suitability in simultaneous saccharification and fermentation. Lactic acid fermentation was optimized at 4 days, pH 5.0, 50°C, 6.0% cellulose of ATDPL, 30 FPU/ g cellulose, 1.0 g. L^-1^ Tween 80, and 5.0 g. L^-l^ yeast extract using *Lactobacillus delbrueckii*. The conversion efficiency of lactic acid from the cellulose of ATDPL was 98.71%, and the lactic acid productivity was 0.719 g. L^-1^ h^-1^. Alkaline treatment exhibited a valuable effect on the production of cellulases and lactic acid by reducing the lignin content and cellulose crystallinity. The results of this study offer a credible procedure for using date palm leaves for microbial industrial applications.

## 1 Introduction

The biological conversion of agricultural wastes is considered to be one of the most pivotal advances concerning the production of valuable products while decreasing pollution issues [1]. The enzymatic turnover of cellulosic wastes is catalyzed by the synergistic action of endoglucanase, exoglucanase, and ß-glucosidase [2]. Thermophilic bacteria are commonly used for cellulase production because of their high cellulolytic activity, shortage generation time, inherent stability of their enzymes, and fast rate of hydrolysis [3, 4]. There is efficient industrial utilization of thermostable cellulase for textiles, paper, detergents, and food industries, as well as the conversion of lignocellulosic wastes to fermentable sugars, which stimulates the search for new sources of these enzymes [2, 4]. The chemical and physical pretreatments of lignocellulosic wastes are more effective due to the complex structure resulting from the relation of cellulose and hemicellulose with lignin. Alkaline pretreatment of agricultural wastes is necessary to remove lignin, decrease the cellulose crystallinity, increase accessible surface area, and enhance the receptivity of the substrates to catalysts [5, 6]. Lactic acid production from renewable wastes by simultaneous saccharification and fermentation (SSF) is a fundamental biotechnological process [7]. As of late, lactic acid has received interest because of the potential market development opportunities for its different applications in the cosmetic, therapeutic, food, leather, chemical feedstock, and textile industries, as well as manufacture of biodegradable plastic, poly-lactic acid [8]. The proficiency and financial aspects of lactic acid production remain actual challenges, for the most part due to the cost of the substrate and batch fermentation in addition to technical problems related to low rates of cellulose hydrolysis due to end-product inhibition [9, 10]. Recently, there have been different endeavors to produce lactic acid efficiently from renewable agriculture wastes: corn straw, rice straw, food waste, corn stover, paper sludge, and sugarcane bagasse [11, 12, 13, 14]. One of the most important agricultural crops in arid and semi-arid regions of the world is the date palm. It is found abundantly in the Arabian Gulf where Saudi Arabia is the world’s third-largest producer of the date palm, with more than 15,000 tons of date palm leaves and trunk viewed as an environmental issue [15, 16]. SSF is viewed as a more appropriate technique for the bioconversion of renewable wastes, where the fermentable sugars delivered by saccharification can be directly fermented into products in the same reactor, lessening feedback inhibition, increasing productivity, and diminishing the fermentation period [17]. The major challenge to the accomplishment of this strategy is the corresponding ideal conditions for enzymatic hydrolysis and growth of fermented microbes [18]. Bacterial strains like *Lactobacillus brevis*, *Lactobacillus helveticus*, and *Bacillus coagulans* can produce lactic acid with complex nutrient necessities and low growth rates, which are the main issues of the industrial production of lactic acid [10, 19]. *Lactobacillus delbrueckii* is a homo-fermentative L-lactic acid-producing bacteria, capable of fermenting glucose by the Embden Meyerhof Parnas pathway [18, 19]. This investigation seeks to uncover a novel bacterial isolate producing cellulase with remarkable properties that might be useful for lactic acid production by SSF utilizing renewable date palm leaves as cellulosic waste.

## 2 Materials and methods

### 2.1 Alkaline treatment and compositional analysis of date palm leaves

Date palm leaves (DPL) were collected from a date palm plantation in Saudi Arabia. They were alkaline pretreated with 2N NaOH at 30°C for 48 h [12]. Then they were washed thoroughly with water and dried before being ground by mechanical milling (Retsch SM100, Haan, Germany) and sieved (Restuch sieve shaker, AS 200, Germany) into a particle size less than 0.25 mm. The cellulose, hemicellulose, and lignin contents in treated and untreated samples were determined using the gravimetric method [20]. The difference between the sample weight before and after treatment of 1 g of Soxhlet extracted dried material with 150 mL of NaOH (500 mol/m3) was the hemicellulose content (%w/w). For lignin content, 3 mL of 72% H_2_SO_4_ was added to 0.3 g of extracted material for 2 h, after that it was autoclaved for 1 h/121°C. The hydrolysates of acid-soluble lignin fraction were absorbance estimated at 320 nm. The acid insoluble lignin was determined by drying the residues at 105°C and incinerating at 575°C in a muffle. The cellulose content (%w/w) was calculated by the difference. The crystalline index of the treated and untreated date palm leaves was analyzed by X-ray diffraction diffractometer (Shimadzu, LabX-XRD-6000, USA) with CuKα (λ¼1.5406 Å) radiation. The crystallinity index (CrI) of cellulose was calculated according the following equation [21]: CrI (%) = [(I002 - I18°) / I002] x 100 %, where I002 is the maximum intensity of the (002) lattice diffraction, and I18° is the intensity diffraction at 18°, 2 ɵ degree.

### 2.2 Isolation and screening of cellulase producing bacteria

Soil samples were obtained from Najran region, Saudi Arabia. The isolation procedure was accomplished by the enrichment of soil samples with 10% DPL in 250 ml Erlenmeyer flasks containing 100 ml of Bushnell Haas medium (BHM). The flasks contain (g. L^−1^): 0.2 MgSO_4_ 7H_2_O, 1.0 K_2_HPO_4_, 1.0 KH_2_PO_4_, 1.0 NH_4_NO_3_, 0.05 FeCl_3_ 6H_2_O, and 0.02 CaCl_2_. The enrichment culture was incubated at 50°C/ 200 rpm in a shaking incubator (Shell Lab, SSI5, USA) for one week, then the culture was serially diluted and plated on nutrient agar medium for 2 days. The purified colonies were screened for their cellulolytic capacity by flooding the plates with 1% Congo red with an observation of a clear zone around the colonies. The potential cellulase producer was selected for further study based on enzymatic activity index, a ratio of the zone of clearance diameter to colony diameter. Enzyme index = Colony diameter + clearance diameter / Colony diameter [22].

### 2.3 Molecular identification and phylogenetic tree construction

The bacterial isolate was incubated for 24 h/200 rpm followed by centrifugation at 10000 rpm/15 min (Hermle, Z 326 K, Germany). QIAamp DNA Mini kit (Qiagen Inc., Valencia, CA) was used for genomic DNA extraction [23]. The amplification and sequencing of 16S rRNA using universal primers were accomplished. The primers of 27 F (5’ CCA GCA GCC GCG GTA ATA CG 3’) and 1492 R (5’ ATC GG(C/T) TAC CTT GTT ACG ACT TC 3’) were applied to amplify the specified gene using the PCR Master Mix kit (TAKARA, Japan). The formed PCR product was migrated through 1.5% agarose gel in a 1x TBE running buffer utilizing an electrophoresis unit. The existence of the PCR product after 30 min was affirmed and shot-utilizing gel documentation (BIORAD, USA). The rest of the item was subsequently submitted to sequencing (Macrogen, Korea). The acquired sequence was aligned and contrasted with the genes saved in GenBank (http://blast.ncbi.nlm.nih.gov/Blast.cgi), followed by accommodation in GenBank with an explicit accession number. The connection between the acquired sequence and the other related sequences was obtained through the construction of a phylogenetic tree utilizing MEGA version 5.0.

### 2.4 Cellulase production

Bacterial culture was inoculated with 2% inoculum (2 x 10^8^ CFU/ml) into a 250-ml Erlenmeyer flask containing 50 ml BHM medium (pH 7.0) supplemented with 2.0% of untreated DPL and alkaline treated date palm leaves (ATDPL) as the sole carbon source. Fermentation was carried out up to 96 h at 50°C/200 rpm. Samples were taken at 12 h intervals and centrifuged at 7000 rpm for 10 min. The cell-free supernatant was used for determination of FPase, CMCase, and β-glucosidase activity [3] and the dry cell weight of bacterial growth was calibrated against the optical density (OD_660_) [24].

### 2.5 Cellulase properties

The effect of pH on the activity of cellulase was determined by assaying the enzyme activity at pH values ranging from 4.0 to 9.0 and the pH stability was investigated in the same pH range for 1 h. The following buffers were used at 50 mM: acetate buffer (pH 4.0-5.0), phosphate buffer (pH 6.08.0), and glycine-NaOH (pH 9.0). The optimal temperature of the cellulase activity was studied at 40-90°C, while thermal stability was investigated after pre-incubation for 1.0 h at temperatures in the same range. The relative activity of cellulase (%) was expressed as the ratio of the enzyme activity at different parameters (pH, temperature) to the activity of the control sample under standard assay conditions [25].

### 2.6 Lactic acid production by SSF

*Lactobacillus delbrueckii* subsp. *lactis* (B. 01357) was obtained freeze-dried from the National Collection of Agriculture and Industrial Microorganisms, Budapest, Hungary. The inoculum was prepared by cultivating a loop of the culture into 50 ml MRS broth in a 250 mL Erlenmeyer flask and incubating at 45°C for 24 h. The 10% (v v^-1^) of inoculum having 10^8^ CFU/mL (0.2 OD_660_) was used as the inoculum for SSF medium. The MRS broth medium contained (g. L^−1^): 10.0 peptone, 10.0 meat extract, 5.0 yeast extract, 20.0 glucose, 1.0 Tween 80, 2.0 K_2_HPO_4_, 5.0 NaOAc, 2.0 tri-ammonium citrate, 0.20 MgSO_4_.7H_2_O, and 0.05 MnSO_4_.4H_2_O [26]. The SSF experiments were conducted in 100-ml static Erlenmeyer flasks, containing 50 ml sterilized medium, and incubated at 50°C. The SSF medium consisted of (g. L^-1^): 60.0 date palm leaves as cellulose, 60.0 yeast extract, 1.67 NaOAc, 1.67 NaPO_3_, 1.0 Mg_2_SO_4_.7H_2_O, 0.05 FeSO_4_.7H_2_O, and 0.05 MnSO_4_.H_2_O (pH 5.0). To prevent acidification, 0.6 g CaCO_3_/1.0 g cellulose was added, and the enzyme concentration of 30 filter paper units (FPU)/g cellulose was used [27]. The fermentation culture was centrifuged (7000 rpm/10 min), and the cell-free supernatant was used for the lactic acid assay. The dry cell weight was calibrated against the optical density (OD660) and based on CFU assay. To optimize the production conditions for lactic acid, the effect of time course (12-120 h), initial pH (4.0-8.0), incubation temperature (40-65°C), substrate concentration (1.09.0%), cellulase concentration (10-60 FPU/g cellulose), Tween 80 concentration (0.0-1.8 g. L^-l^), and yeast extract concentration (1.0-8.0 g. L^-l^) were investigated. All experiments were performed in three replicates. The productivity of lactic acid (g. L^-l^ h^-1^) = concentration of lactic acid (g. L^-l^) / fermentation time (h). The conversion efficiency of lactic acid from cellulose (%) was calculated as the concentration of lactic acid produced (g. L^-1^) × 100/ initial concentration of cellulose in the date palm residue (g. L^-l^).

### 2.7 Analytical methods

#### 2.7.1 FPase activity

FPase activity was measured according to Mandels et al. [27]. The reaction mixture contained 0.5 ml of 50 mM acetate buffer at pH 5.0, 0.5 ml culture filtrate, and one filter paper strip (1×3 cm) that was used as the crystalline cellulose substrate. One unit of enzyme activity was defined as the amount of enzyme that released 1 μmol glucose per minute during the reaction.

#### 2.7.2 CMCase activity

CMCase activity was measured as described in the method mentioned above for FPase activity, except that 0.5 ml of 1% carboxymethylcellulose solubilized in 50 mM acetate buffer, pH 4.8, was used as the substrate. One unit of enzyme activity was defined as the amount of enzyme that released 1 μmol glucose per minute during the reaction.

#### 2.7.3 β-Glucosidase activity

Glucosidase activity was assayed with 0.02% p-nitrophenyl-β-D-glucopyranoside as the substrate, in 50 mM acetate buffer, pH 5.0. One unit of enzyme activity was defined as the amount of enzyme required to release 1.0 μM p-nitrophenol per minute during the reaction [28].

#### 2.7.4 Glucose assay

The glucose was estimated by glucose estimation kit (K606-100) provided by Bio Vision Company and quantified (g. L^-1^) from a glucose standard curve.

#### 2.7.5 Lactic acid estimation

For lactic acid assay, 3 mL H_2_SO_4_ (96%) was added to 0.5 mL of sample and incubated at 95-100°C/ 10 min in a steam-water bath. A 50-μL aliquot of CuSO_4_ reagent and 100 μL p-phenyl phenol reagent were added. The absorbance was estimated using a double beam UV/Vis scanning spectrophotometer (Shimadzu, 1601PC, Japan) at 570 nm. For a standard curve, 0-30 μg pure lactic acid was used and the concentration was expressed as g. L^-1^ [30].

### 2.8 Statistical analysis

The ANOVA was carried out for statistical analysis of results and the significant differences at *P*≤ 0.05 were assayed utilizing Minitab (version 15). Values followed by different letters are significantly different. The standard error of the mean for n=3 was represented by the error bars.

## 3 Results

### 3.1 Chemical analysis of date palm leaves

**Figure 1:**
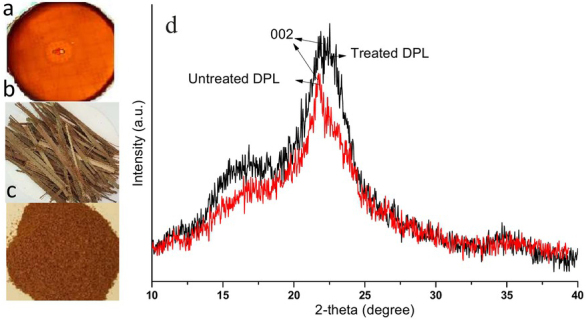
Cellulose hydrolysis (a), Untreated DPL (b), Alkaline treated DPL (c), and X-ray diffractometer pattern for untreated and alkaline treated DPL (d).

Untreated date palm leaves contained 57.13% cellulose, 14.73% hemicellulose, and 18.11% lignin while ATDPL had 68.66% cellulose, 11.5% hemicelluloses, and 2.9% lignin (Table 1). Alkaline pretreatment was significantly diminished the lignin content where about 83.98% of the lignin was removed, in addition to an expansion in the cellulose content up to 12.67% and partial degradation of the hemicellulose by 3.23%. The reduction in the crystallinity index by alkaline pretreatment was shown in the diffractogram from the X-ray analysis (Fig.1d &Table 1). It was apparent that the NaOH-treatment increase the amorphous ratio of cellulose by reducing the crystallinity index from 67% to 56%.

**Table 1:** Chemical composition of untreated and NaOH-treated date palm leaves

Date palm leaves	Chemical composition (%)			
	Cellulose	Lignin	Hemicellulose	CrI
Untreated	57.17±0.59	18.11±0.48	14.73±0.49	67
NaOH-treatment	68.66±0.39	2.90±0.35	11.50±0.48	56

(CrI) crystallinity index, (±) Standard error of the mean for n= 3.

### 3.2 Isolation, screening and molecular identification of cellulase producing bacteria

**Figure 2:**
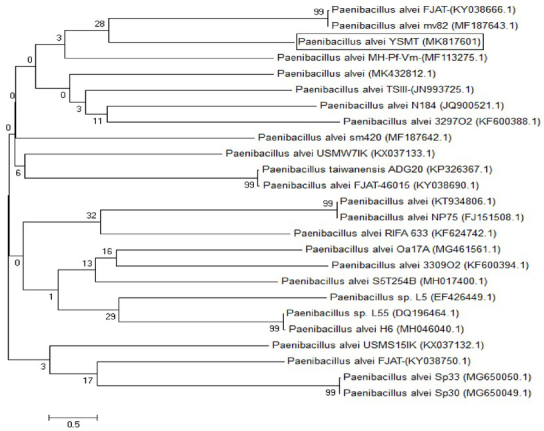
Phylogenetic relationship of *Paenibacillus alvei* and other related 16S rRNA gene sequences of published strains generated by the Neighbor-Joining method using MEGA 5.0 software.

Twenty-one bacterial isolates were isolated from soil samples enriched with date palm leaves and screened for their capacity to produce cellulase utilizing the Congo red test (Figure 1b). The bacterial isolate demonstrating the highest enzyme activity index (3.2 ± 0.10) was chosen as a promising cellulase producing isolate. The molecular identification of the isolate depended on the amplification and sequencing of 16S rRNA. The amplified sequence revealed that the isolate is highly similar to *Paenibacillus alvei* with a 99% similarity percentage. The sequence of the isolate was deposited in GenBank with accession number MK817601. The phylogenetic relationship of the *Paenibacillus alvei* YSMT and the other related species in GenBank database was constructed as shown in Figure 2.

### 3.3 Production of cellulase using untreated DPL and alkaline treated DPL

**Figure 3:**
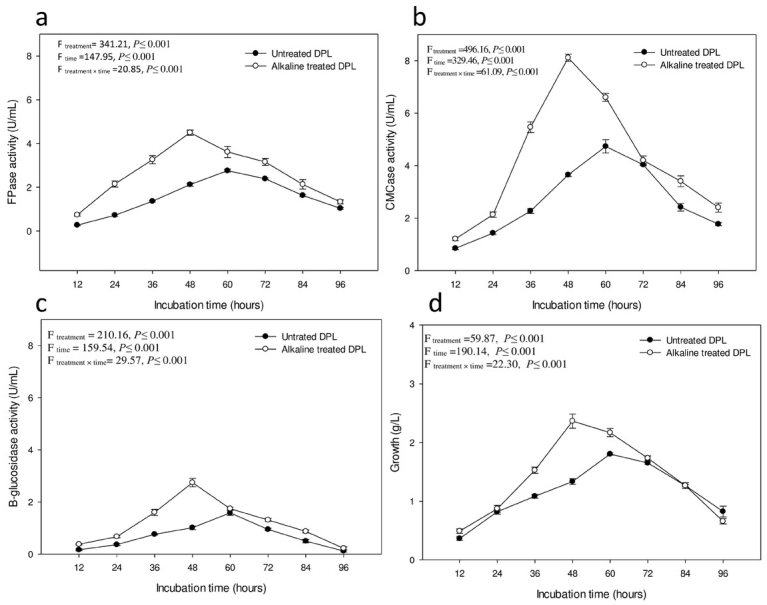
Production of cellulase by *Paenibacillus alvei* using untreated and alkaline treated DPL. FPase (a), CMCase (b), β-glucosidase (c) and bacterial growth (d).

ATDPL was the best inducer for cellulase production when compared with untreated leaves (Figure 3 a,b,c). It was found that the most noteworthy increment of cellulase production using ATDPL was acquired after 48 h of incubation and yielded 63.04% FPase, 71.45% CMCase, and 73.41% β-glucosidase. Maximum cellulase production was consistent with the growth pattern where the production of the enzyme was low during the lag phase to the inception of the logarithmic phase of growth, while the peak of enzyme production occurred at the beginning of the stationary phase after 48 h and 60 h for treated and untreated leaves, respectively (Figure 3d). These observations show that the alkaline treatment was valuable for improving its susceptibility to enzymatic hydrolysis and bacterial growth.

### 3.4 Cellulase properties

**Figure 4:**
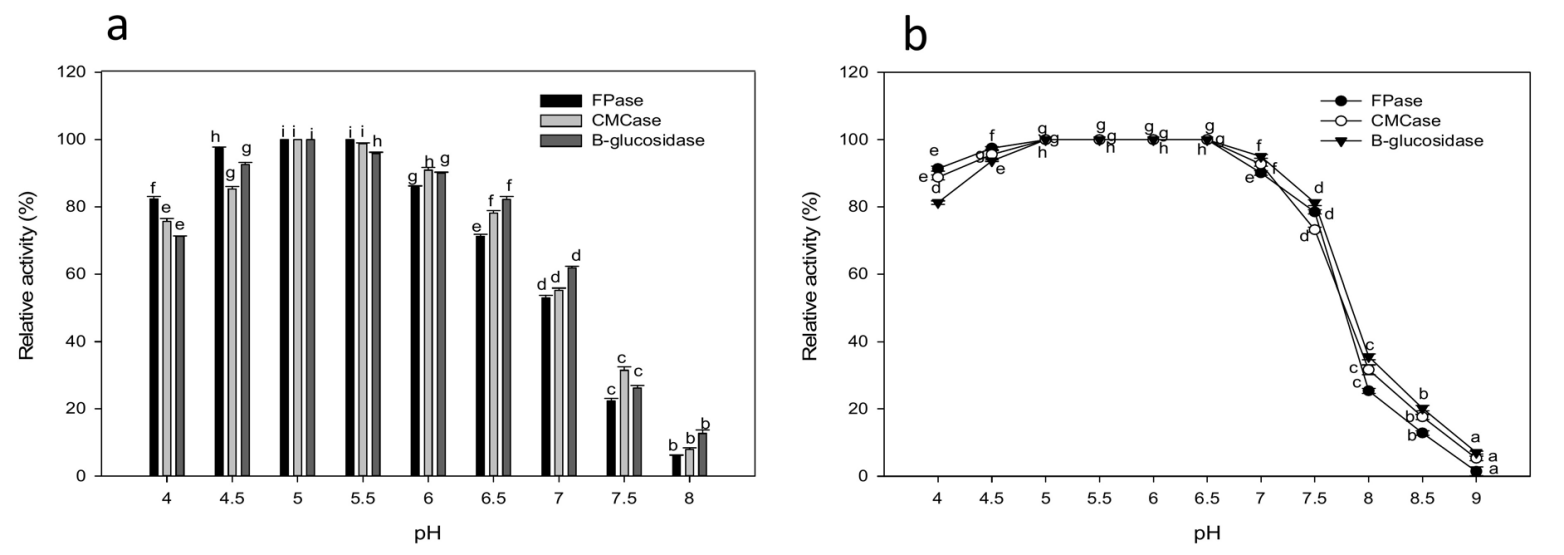
Effect of pH on cellulase activity (a) and stability (b). Values followed by different letters are significantly different..

**Figure 5:**
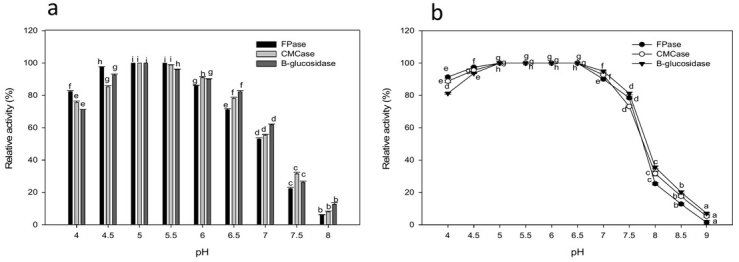
Effect of temperature on cellulase activity (a) and stability (b). Values followed by different letters are significantly different.

Cellulase activity was most efficient in the acidic pH range from 4.0 to 6.0, with highest efficiency at pH 5.05.5. Beyond the optimal pH levels, an extreme decrease in enzyme activity was observed (Figure 4). Enzyme stability was observed in the pH range of 4.0-7.0 after pre-incubation in buffers of various pH for 1 h with complete stability at pH 5-6.5. The sharp drop in pH stability at pH 8.0 reached 25.4%, 31.66% and 35.56% for FPase, CMCase, and β-glucosidase, respectively. Cellulase activity was highest between 45 and 60°C, with maximum activity at 50°C (Figure 5). About 91.06% FPase, 94.53% CMCase, and 98.44% β-glucosidase activity was retained at 60°C. Enzyme stability remains unaffected after thermal treatment from 40 to 65°C while retaining 97.8%, 95.76%, and 92.56% of the initial activity of FPase, CMCase, and β-glucosidase after treatment at 70°C for 1 h, respectively.

A progressively adverse effect on stability was found by pretreating the enzymes at 90°C.

### 3.5 Lactic Acid Production by SSF

#### 3.5.1 Effect of time course

**Figure 6:**
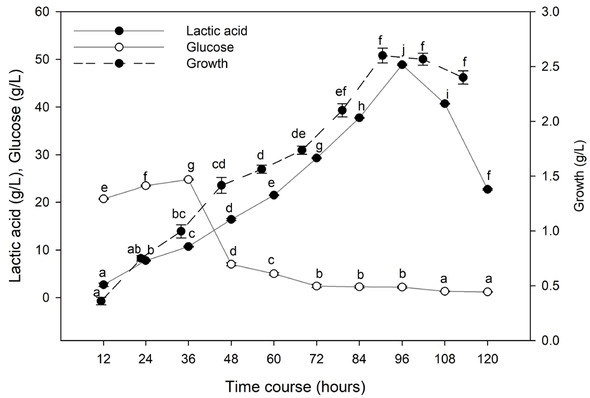
Effect of time course on lactic acid production by SSF. Values followed by different letters are significantly different. The rate of lactic acid fermentation was monitored at 5.0 pH, 50°C, 6% substrate, 30 FPU/g cellulose, and 6.0 g. L^-l^ yeast extract conc.

Lactic acid produced by *L. delbrueckii* subsp. *lactis* using ATDPL as a cellulosic source was maximally achieved (48.85 g. L^-1^) after 96 h of incubation at the beginning of a stationary phase of bacterial growth (Figure 6). The amount of glucose which is continuously produced and simultaneously converted by *L. delbrueckii* to lactic acid was sufficient to sustain bacterial growth. The conversion efficiency of lactic acid and the productivity were 81.4% and 0.508 g. L^-1^h^-1^, respectively. Further increasing the fermentation period did not result in a significant increase in lactic acid production. The rate-limiting step for lactic acid production was enzymatic hydrolysis since the glucose concentration was very low throughout the SSF. It increased rapidly at early stages of SSF up to around 36 h, and thereafter gradually declined and remained constant.

#### 3.5.2 Effect of initial pH

**Figure 7:**
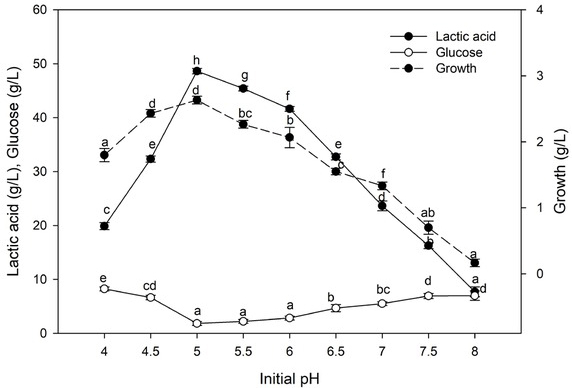
Effect of initial pH on lactic acid production. Values followed by different letters are significantly different. The rate of lactic acid fermentation was monitored at 96 h, 50°C, 6% substrate, 30 FPU/g cellulose, and 6.0 g. L^-l^ yeast extract conc.

The results demonstrated that pH 5.0 was the most conducive to lactic acid production as well as cellulase activity (Figure 7). Moreover, the abatement in lactic acid production under acidic condition was low when contrasted with alkaline conditions. Lactic acid production reached about 85.60% at pH 6.0 while, at pH 8.0, it sharply decreased to 15.83%.

#### 3.5.3 Effect of incubation temperature

**Figure 8:**
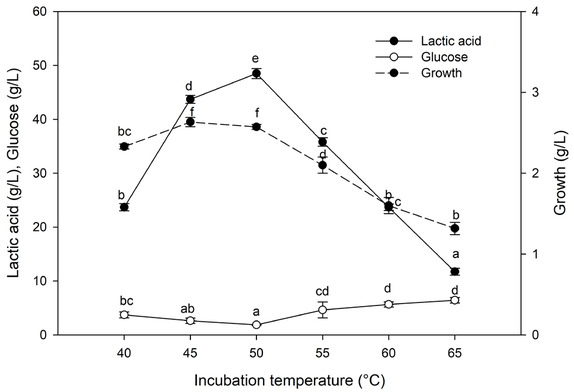
Effect of incubation temperature on lactic acid production. Values followed by different letters are significantly different. The rate of lactic acid fermentation was monitored at 96 h, 5.0 pH, 6% substrate, 30 FPU/g cellulose, and 6.0 g. L^-l^ yeast extract conc.

Lactic acid production was extraordinarily influenced by incubation temperature (Figure 8). Highest concentrations were seen in the temperature range of 45-50°C while the lowest concentration was observed at 65°C. There was an increase in glucose consumption as the temperature rose from 40 to 45°C, and a further increase to 50°C brought a slight improvement in the fermentation process.

#### 3.5.4 Effect of substrate concentration

**Figure 9:**
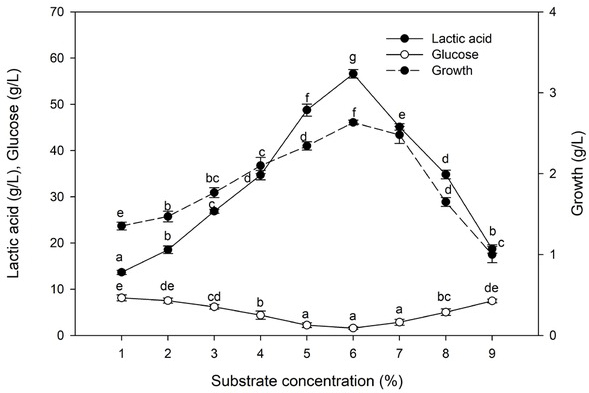
Effect of substrate concentration on lactic acid production. Values followed by different letters are significantly different. The rate of lactic acid fermentation was monitored at 96 h, 5.0 pH, 50°C, 30 FPU/g cellulose, and 6.0 g. L^-l^ yeast extract conc.

The rate of enzymatic hydrolysis of cellulose and fermentation is immediately proportional to cellulose concentration up to the ideal level (Figure 9). The most significant increase in lactic acid production was acquired with 7.0% cellulose of ATDPL reaching 56.6 g. L^-1^. Further increasing the substrate amount to 10% decreased the productivity to 18.7 g. L^-1^. Glucose concentration was minimal at the optimal substrate concentration, which promotes the concept that enzymatic hydrolysis is the rate-limiting step for SSF.

#### 3.5.5 Effect of cellulase concentration

**Figure 10:**
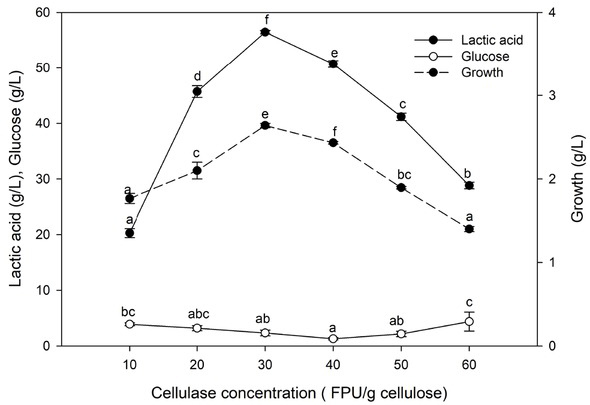
Effect of cellulase concentration on lactic acid production. Values followed by different letters are significantly different. The rate of lactic acid fermentation was monitored at 96 h, 5.0 pH, 50°C, 6% substrate, and 6.0 g. L^-l^ yeast extract conc.

Lactic acid production depended emphatically on cellulase concentration (Figure 10). It continuously increased up to 30 FPU/g cellulose. A decrease in sugar depletion was seen at high cellulase concentrations, evidence of undesirable conditions for lactic acid fermentation by SSF. An increase in enzyme concentration to 40 FPU/g substrate diminished the production rate by 25.46%.

#### 3.5.6 Effect of surfactant Tween 80

**Figure 11:**
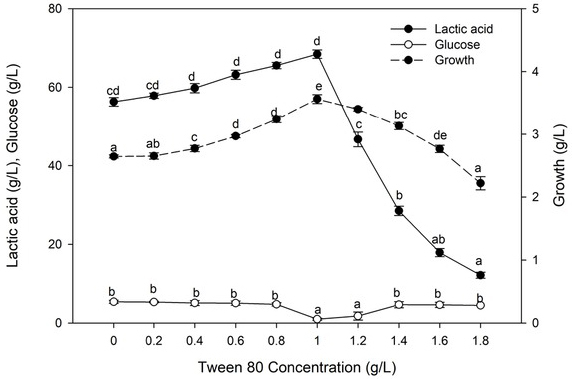
Effect of Tween 80 on lactic acid production. Values followed by different letters are significantly different. The rate of lactic acid fermentation was monitored at 96 h, 5.0 pH, 50°C, 6% substrate, 30 FPU/g cellulose, and 6.0 g. L^-l^ yeast extract conc.

Lactic acid production and growth rate gradually increased in progress with an increase in Tween 80 content. Significant lactic acid production (68.45 g. L^-1^) was accomplished at 1.0 g. L^-1^ Tween 80 and raised the production ratio by 21.58% (Figure 11). It was observed that at a high concentration of Tween 80, there was high abatement in the lactic acid produced reaching 17.72% at 1.8 g. L^-1^ Tween 80.

#### 3.5.7 Effect of yeast extract concentration

**Figure 12:**
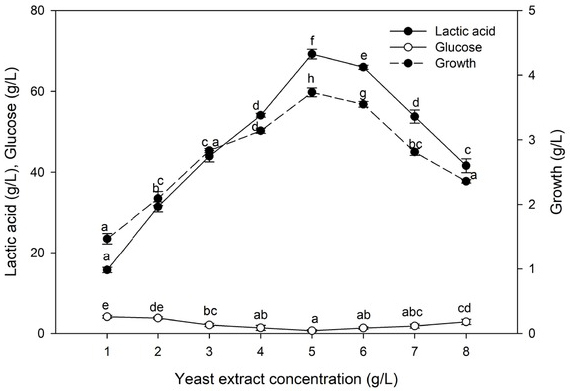
Effect of yeast extract on lactic acid production. Values followed by different letters are significantly different. The rate of lactic acid fermentation was monitored at 96 h, 5.0 pH, 50°C, 6% substrate, 30 FPU/g cellulose, and 1.0 g. L^-l^ Tween 80.

A crucial boost in lactic acid production was observed at 5% yeast extract (Figure 12). At non-ideal yeast extract values, lactic acid production was diminished by 21.85% and 39.86% at 4.0% and 8.0%, respectively. Furthermore, lactic acid production was associated with growth, where the optimal growth rate was at 5% yeast extract. Under optimal conditions, the conversion efficiency of ATDPL cellulose after optimized SSF process was 98.71% and the lactic acid productivity was 0.719 g. L^-1^ h^-1^.

## 4 Discussion

Integrated bioprocessing is a cost-effective approach for the conversion of lignocellulosic wastes to important bio-chemicals by achieving microbial cellulase production, pretreatment of lignocellulosic materials, saccharification, and fermentation [31]. The chemical analysis of untreated DPL and alkaline treated DPL revealed that alkaline pretreatment was valuable for diminishing the lignin content by removing about 83.98% with a slight decrease in hemicellulose and increase in cellulose content. These outcomes were compatible with results reported previously [6, 32, 33]. Alkaline treatment of lignocellulosic wastes is the fundamental mechanism of degradation of lignin structure; it expands the inward surface area, diminishes the level of polymerization and crystallinity, separates structural linkages among lignin and carbohydrates, and disrupts lignin structure [33]. The increase in cellulose content by alkaline treatment is due to the disruption of its ultrastructure and the linkage among the lignocellulosic components [34]. The relatively amorphous structure of hemicellulose maybe explains its loss by depolymerization of hetero-polysaccharides in alkaline pretreatment [33, 34]. The reduction in crystallinity structure of cellulose by alkaline pretreatment was analyzed by X-ray diffractometer (Figure 1d). The de-crystallization of cellulose by alkaline treatment was achieved by alterations in the molecular and supramolecular cellulose structure forming a more amorphous structure [35, 36]. The NaOH treatment acts as a swelling agent that was able to penetrate the crystalline region of cellulose by disrupting hydrogen bonds [34].

Thermophilic bacteria represent an appealing potential source of cellulase due to their rapid growth rate, enzyme complexity, and extreme habitat variability [37]. The promising cellulase producer *Paenibacillus alvei* was isolated from soil samples and produces a mixture of FPase, CMCase and β-glucosidase. It was obvious that the enhancement of bacterial growth and cellulase production using ATDPL relative to untreated samples was due to the reduction of lignin content (Table 1) and crystallinity index (Figure 1). Actually, the natural fiber of date palm trees exhibited a higher crystallinity index: date palm trunk 69.2%, leaf stalk 73.7%, leaf sheath 72.4%, and fruit bunch stalk 78.7% [5]. In this way, pretreatment of lignocellulosic wastes is fundamental for efficient use [32, 33]. Alkaline treatment was useful for improving susceptibility to enzymatic hydrolysis and bacterial growth. It was recently reported that alkaline treatment wheat straw, rice straw, and bagasse were more prone to the enzymatic hydrolysis by *Bacillus subtilis* cellulase than untreated materials [38]. The changes in chemical nature as well as the increase in the amorphous fraction and nutrient availability of the treated materials led to enhanced enzymatic digestibility and bacterial growth [5, 36]. To our knowledge, no study has reported on cellulase production by and applications of *Paenibacillus alvei*, though other species of *Paenibacillus* have been reported to be cellulase producers which are able to hydrolysate many lignocellulosic wastes: *P. terrae*, *P. polymyxa*, *P. barcinonensis*, *P. xylanilyticus* KJ03, *Paenibacillus* sp. CKS1, *P. cookii* SS-24, and *P. dendritiformis* [3, 4, 39, 40]. Thus, further studies are required to confirm the efficiency of *Paenibacillus alvei* cellulase against different agricultural residues depending on the results of the present study.

The characterization of *P. alvei* cellulase in regard to pH and temperature activity and stability demonstrated that this enzyme has the greatest proficiency in conditions similar to the SSF procedure. Its cellulase showed a noteworthy increase in activity at 50-55°C and stability at 40-70°C in addition to exhibiting highly significant activity at pH 5.0-5.5 and remaining stable within a pH range of 4.0-7.0. A similar pattern of results was observed for *Paenibacillus* strains, where ideal pH and temperature for CMCase activity produced by *Paenibacillus terrae* ME27-1 were 5.5 and 50°C, respectively, and the enzyme was stable at a wide pH range of 5.0-9.5 [3] while, the CMCase activity of *Paenibacillus* sp. CKS1 was optimal at 50 °C and pH 4.8 [39]. Additionally, optimal pH and temperature for cellulose hydrolysis by *P. dendritiformis* KFX-0 cellulase were 6.0 and 55°C, respectively [4]. At pH values beyond the ideal level, enzyme activity was affected because of the change in conformation of the enzyme’s active site, a consequence determined by the ionic and hydrogen bonding, which can be influenced by pH [31]. Thermostable cellulolytic enzymes have numerous points of interest: they are utilized in fermentation processes at raised temperatures, forestall microbial contamination, expand reaction rates due to a decrease in viscosity, and increase the diffusion coefficient of substrates, as well as enhancing process yield, owing to the increased solubility of the substrates and products [41].

Lactic acid is a valuable organic acid widely utilized in chemical feedstock, pharmaceuticals, food, as well as in the production of polylactic acid, an environmentally-friendly alternative to plastic [7]. The main purpose of this study was to analyze the production of lactic acid by *L. delbruckii* using ATDPL as the cellulosic source and *P. alvei* cellulase. Maximum lactic acid production was accomplished after 96 h of incubation, which brought an increase in the conversion efficiency and lactic acid productivity to 81.4% and 0.508 g. L^-1^ h^-1^. The drop in lactic acid production in other than ideal conditions might be due to the hindrance of bacterial growth by organic acids, which is related to the solubility of the non-dissociated form inside the cytoplasm and the insolubility of the ionized acid form [18]. The acidity of the cytoplasm of the microbial cell and the collapse of the proton motive force constrain nutrients transport [42]. The high glucose content at the beginning of the fermentation period and, after that, its continuous lessening can be explained by the non-acclimatization of microbes to the nature of SSF causing the decline of their metabolism [15]. These observations were comparable with those of Abe and Takogi [26], who found that ideal lactic acid production by SSF was achieved after incubation for 50 h and 120 h, using milled newspaper and cellulose powder as cellulosic substrates, respectively, while Parajo et al. [43] observed highest lactic acid production of 30 g. mL^-1^ at 60 h of fermentation. Conversely, according to certain researchers, the optimal period for lactic acid production by *L. delbruckii* was between 4.0 and 7.0 days [44]. Likewise, 55.6 g. L^-1^ of lactic acid was produced by SSF of corn cob waste using *L. delbrueckii* ZU-S2 [45]. However, the main obstacle of SSF is that the optimal conditions for the saccharification and fermentation procedures ought to be identical. Hence, the factors influencing lactic acid production by the SSF strategy were researched.

pH is one of the most crucial operational factors affecting lactic acid production by SSF. Our results showed that pH 5.0-5.5 was the most ideal for lactic acid production as well as for cellulase activity. The findings concurred with numerous searchers [27, 46], who described pH 5.0 as preferential for lactic acid production by SSF. In contrast, other studies noted that maximum lactic acid production was observed at pH 6.0 from food wastes and corn stover by *L. delbrueckii* and *B. coagulans*, respectively [18, 47]. likewise, Idris and Suzana [48] demonstrated that *L. delbrueckii* grows well at an initial pH in the region of 5.0-7.0, and their lactic acid production sharply decreased at high pH as a result of severe stress on the organism’s metabolic abilities.

The results delineating the impact of incubation temperature on lactic acid production were predictable in light of the fact that 45-50°C was the ideal temperature for the growth of *L. delbrueckii*, which is close to the optimal temperature for cellulase activity. These results confirmed what was reported in previous studies that mentioned to 45°C as the optimum temperature for lactic acid production by SSF [43]. Other results indicated that at 37°C, the highest concentrations of lactic acid were produced by *L. delbrueckii* using pineapple waste. This disparity may be explained by diverse substrates and nutrients utilized in the lactic acid fermentation process [48].

A significant increase in lactic acid production (56.6 g. L^-1^) was observed with ATDPL at 7.0% cellulose. These results contrast with Cheng et al. [49], who reported that only 15.2 g. L^-1^ lactic acid was produced from corn stover by SSF at 30 g. L^-1^ cellulose using *Lactococcus lactis* subsp. *lactis* ATCC 11454. Numerous studies found different ideal substrate levels for lactic acid production by SSF including 6% rice straw [46], 15% empty fruit bunch [50], and 14.4% alkaline-pretreated corn stover [17]. Diminishing lactic acid production with higher substrate concentration is due to an increase in the osmotic pressure caused by the high sugar content and an increase in the medium viscosity [46]. The high viscosity of the medium impeded the blending procedure, and it resulted in poor availability of the enzymes to the substrate and a high concentration of inhibitors that reduce cell growth and metabolism [48]. This result affirmed the thought that saccharification is the rate-limiting step for lactic acid production [27].

The cost of cellulase is a significant impediment to the bioconversion of lignocellulosic wastes [44]. The optimal cellulase concentration for lactic acid production was 30 FPU/g cellulose. At this value, the maximum rate of enzymatic hydrolysis of alkaline treated DPL to glucose was achieved, and the glucose was immediately converted by L. delbrueckii to lactic acid. Lactic acid production from sweet sorghum bagasse through SSF using *Lactobacillus lactis* was optimal at a cellulase loading of 25 FPU/g substrate [51]. Additionally, maximum lactic acid production by SSF of empty fruit bunch was observed at a cellulase concentration of 50 FPU/g cellulose and at a high concentration, bacterial growth was quelled because of the high sugar concentration aggregated during the SSF procedure [52].

A positive impact of surfactant Tween 80 on lactic acid production was observed at 1.0%. Tween 80, which able to dissolve lipid structures in the cell membrane, thereby improving membrane permeability and increasing the release of metabolites [2, 53]. Furthermore, surfactants may alter the nature of substrates by increasing the available cellulose surface or by expelling inhibitory lignin. They likewise improve the stability of enzymes and thereby diminish enzyme denaturation during hydrolysis [54]. At high concentrations, however, lactic acid production was decreased, as the toxicity of Tween 80 results in destruction and loss of functionality of the cell membrane by solubilization the lipid bilayer [55].

Lactic acid bacteria require an abnormal level of nutrients, including vitamins, minerals, and amino acids, and so yeast extract has been commonly utilized as a nitrogen source since it outcompetes other nitrogen sources and contains multi-nutrients for lactic acid production [56]. The efficacy of converting the cellulose of treated date palm leaves into lactic acid was raised to 98.71% with a productivity value 0.719 g. L^-1^ h^-1^ at 5% yeast extract. Our results agreed with Yao et al. [46]. It is noticeable that this concentration was half the amount previously published by numerous investigators [17, 18, 49], who expressed that yeast extract at 10 g.L^-1^ was an appropriate nitrogen source for maximal lactic acid production when almost no sugar was left in the fermentation medium. The presence of nitrogen in the date palm residue where the crude protein concentration of date leaves is about 10.6-16.5% may explain these results [57].

## 5 Conclusion

Lignocellulosic wastes of the date palm are abundant in the Arabian Gulf and, in particular, Saudi Arabia. They can be utilized effectively for cellulase production and lactic acid biosynthesis. Maximum cellulase production was obtained by the bacterial isolate *Paenibacillus alvei* using alkaline treated DPL as a sole carbon source after 48 h. Ideal enzyme conditions have agreed with conditions preferential for lactic acid production by SSF. The optimal lactic acid production was accomplished after incubation for 96 h at an initial pH of 5.0, 50°C, 6% cellulose substrate, 30 FPU/g cellulose, 1.0 g. L^-1^ Tween 80, and 5 g. L^-1^ yeast extract. The conversion efficiency of lactic acid from cellulose was 98.71%, and the lactic acid productivity was 0.719 g. L^-1^ h^-1^.
