# Erratum to “Thermostable cellulase biosynthesis from *Paenibacillus alvei* and its utilization in lactic acid production by simultaneous saccharification and fermentation”

**DOI:** 10.1515/biol-2020-0104

**Published:** 2020-10-28

**Authors:** Yasser S. Mostafa, Saad A. Alamri, Mohamed Hashem, Nivien A. Nafady, Kamal A. M. Abo-Elyousr, Zakaria A. Mohamed

In the published article “Mostafa YS, Alamri SA, Hashem M, Nafady NA, Abo-Elyousr KAM, Mohamed ZA. Thermostable cellulase biosynthesis from *Paenibacillus alvei* and its utilization in lactic acid production by simultaneous saccharification and fermentation. Open Life Sci. 2020;15:185–97. doi: 10.1515/biol-2020-0019” the affiliations of authors Kamal A. M. Abo-Elyousr and Zakaria A. Mohamed are incorrect.

The correct affiliations of Kamal A. M. Abo-Elyousr and Zakaria A. Mohamed are as follows:

**Kamal A. M. Abo-Elyousr**, Assiut University, Faculty of Agriculture, Plant pathology Department, Assiut, Egypt; King Abdulaziz University, Faculty of Meteorology, Environmental and Arid Land Agriculture, Department of Arid Land Agriculture, Saudi Arabia

**Zakaria A. Mohamed**, Department of Botany & Microbiology, Faculty of Science, Sohag University, Sohag, 82524, Egypt

